# Transcriptional Activity and Nuclear Localization of Cabut, the *Drosophila* Ortholog of Vertebrate TGF-β-Inducible Early-Response Gene (TIEG) Proteins

**DOI:** 10.1371/journal.pone.0032004

**Published:** 2012-02-16

**Authors:** Yaiza Belacortu, Ron Weiss, Sebastian Kadener, Nuria Paricio

**Affiliations:** 1 Departamento de Genética, Facultad CC Biológicas, Universidad de Valencia, Burjasot, Spain; 2 Department of Biological Chemistry, The Alexander Silberman Institute of Life Sciences, Hebrew University of Jerusalem, Edmond J. Safra Campus, Givat-Ram, Jerusalem, Israel; University of Massachusetts Medical School, United States of America

## Abstract

**Background:**

Cabut (Cbt) is a C_2_H_2_-class zinc finger transcription factor involved in embryonic dorsal closure, epithelial regeneration and other developmental processes in *Drosophila melanogaster*. Cbt orthologs have been identified in other *Drosophila* species and insects as well as in vertebrates. Indeed, Cbt is the *Drosophila* ortholog of the group of vertebrate proteins encoded by the TGF-ß-inducible early-response genes (TIEGs), which belong to Sp1-like/Krüppel-like family of transcription factors. Several functional domains involved in transcriptional control and subcellular localization have been identified in the vertebrate TIEGs. However, little is known of whether these domains and functions are also conserved in the Cbt protein.

**Methodology/Principal Findings:**

To determine the transcriptional regulatory activity of the *Drosophila* Cbt protein, we performed Gal4-based luciferase assays in S2 cells and showed that Cbt is a transcriptional repressor and able to regulate its own expression. Truncated forms of Cbt were then generated to identify its functional domains. This analysis revealed a sequence similar to the mSin3A-interacting repressor domain found in vertebrate TIEGs, although located in a different part of the Cbt protein. Using β-Galactosidase and eGFP fusion proteins, we also showed that Cbt contains the bipartite nuclear localization signal (NLS) previously identified in TIEG proteins, although it is non-functional in insect cells. Instead, a monopartite NLS, located at the amino terminus of the protein and conserved across insects, is functional in *Drosophila* S2 and *Spodoptera exigua* Sec301 cells. Last but not least, genetic interaction and immunohistochemical assays suggested that Cbt nuclear import is mediated by Importin-α2.

**Conclusions/Significance:**

Our results constitute the first characterization of the molecular mechanisms of Cbt-mediated transcriptional control as well as of Cbt nuclear import, and demonstrate the existence of similarities and differences in both aspects of Cbt function between the insect and the vertebrate TIEG proteins.

## Introduction

*cabut* (*cbt*) encodes a *Drosophila* transcription factor (TF) containing three C_2_H_2_ zinc finger motifs at the carboxy (C) terminus and a serine-rich (SR) region at the amino (N) terminus [1]. This protein is involved in dorsal closure during *Drosophila* embryogenesis [1], but it is also required for other developmental processes such as the ecdysone response [2], neuroendocrine cell remodeling [3], epithelial regeneration [4], circadian rhythms [5], axon guidance and synaptogenesis [6], [7], pole cell formation [8], cell growth [9], [10], autophagic cell death [11], cell cycle progression (A.J. Katzaroff and E.A. Bruce, personal communication) and cell proliferation and patterning [12]. Experiments in *Drosophila* embryos and S2 cells have shown that Cbt is a nuclear protein, although it is also present in axons in the central and peripheral nervous systems [13]. Cbt orthologs have been identified in other *Drosophila* species and insects, including the mosquito (*Anopheles gambiae* and *Aedes aegypti*), red flour beetle (*Tribolium castaneum*), honeybee (*Apis mellifera*) and silkworm (*Bombyx mori*), as well as in other invertebrate organisms such as ascidians, echinoderms and crustaceans [14], [15]. The expression patterns of *cbt* transcripts and proteins during embryonic development are highly conserved among Drosophilidae [13], [14]. Interestingly, Cbt also presents high similarity to the vertebrate proteins encoded by the TGF-β-inducible early-response genes (TIEGs) [14], [16] and has also been named *Drosophila TIEG* (*dTIEG*) [12].

TIEG proteins belong to subgroup III of the Sp1-like/Krüppel-like family of TFs, which contain three highly conserved C-terminal C_2_H_2_-type zinc finger motifs that mediate binding to GC-rich promoter sequences [17], [18], [19], [20], [21]. Their expression is regulated by a plethora of growth factors (e.g., TGF-ß superfamily), cytokines (e.g., BMP family and activin A) and hormones (e.g., estrogens) (reviewed in [20]). Several proteins of this family have been characterized, including TIEG1 (Krüppel-like factor 10, KLF10) and TIEG3 (KLF11) in humans and mice, and and TIEG2/3 (KLF11) in mice (17,19,20,21, reviewed in [18]). They have been also identified in rat, monkey, pig and zebrafish genomes [14], [22]. TIEG proteins are involved in numerous processes, including, among others, proliferation, apoptosis, differentiation, cancer and circadian rhythms [17], [23], [24], [25], [26], [27], [28], [29], [30]. These proteins can function as either transcriptional repressors [31], [32] or activators [32], [33], [34], [35], depending on the cellular context, the promoter to which they bind and the coregulators with which they interact [33], [34], [35]. Several studies have identified and characterized functional domains in TIEG proteins. One proline-rich (PR) region and three repression domains (R1, R2 and R3) were identified at the N-terminal region of TIEG proteins [19], [31]. Interestingly, a mammalian mSin3A-interacting domain (SID) was identified in the R1 domain of the TIEG3 protein and shown to be essential for TIEG3-mediated transcriptional repression in cell culture [31], [32]. This domain interacts with the co-repressor mSin3A, which inhibits transcriptional activation of target genes by histone deacetylation and subsequent remodeling of chromatin structure [36]. Different sequences are found in the R2 and R3 domains [31]. Moreover, the C-terminal end of TIEG3 contains the DNA-binding domain (DBD) and an additional downstream domain, both of which are able to activate transcription in OLI-neu and HeLa cells [31], [32]. More recently, other domains involved in transcriptional regulation have been identified in TIEG proteins, including an N-terminal domain in TIEG1 which interacts with the enzyme Jumonji AT-rich interactive domain 1B/lysine-specific demethylase 5B (JARID1B/KDM5B) and mediates transcriptional repression [37], and a C-terminal domain in TIEG2 that interacts with the p300 co-activator to activate expression of the *Pdx1* gene [33]. Regarding their nuclear localization, TIEGs and other KLF proteins, contain a bipartite NLS within the zinc finger domains, that is required for transport to the nucleus [32], [38], [39]. In general, NLSs consist of either one (monopartite) or two (bipartite) stretches of basic amino acids (usually arginine (R) and lysine (K)) separated by an intervening region of 10–12 residues and recognized by protein carriers called importins [40]. The NLSs frequently overlap with DBDs [41], as occurs in the TIEG3 protein [32]. Because nuclear transport of TFs is essential for cellular function, regulation of TF nuclear availability through NLSs directly affects gene expression, cell growth and proliferation [42].

Cbt is the *Drosophila* ortholog of vertebrate TIEG proteins [14], [16] and shares functions with several family members, e.g., rat TIEG1 and murine TIEG3, as it is involved in circadian rhythms as well as cell proliferation and positive regulation of TGF-β signaling [5], [12]. Regarding its transcriptional activity, previous results suggested that Cbt may function as an activator of gene expression. We showed that *decapentaplegic* (*dpp*) expression was downregulated at the leading edge of the lateral epidermal sheets during dorsal closure in *cbt* mutant embryos [1]. Cbt also positively regulates the expression of *STAT92E*, *spalt* (*sal*) and *optomotorblind* (*omb*) genes in wing imaginal discs [12]. Although these results suggest that Cbt may activate gene expression in embryos and wing discs, it is not clear whether the transcriptional regulation of these target genes is direct or indirect. In the present study, we performed a functional characterization of the Cbt protein by examining its transcriptional regulatory potential and identifying its functional domains. Gal4-based transcriptional assays in S2 cells demonstrated that Cbt is a transcriptional repressor and contains a SID similar to the one identified in TIEG proteins [31], [32]. We also report that Cbt can downregulate its own expression, probably by directly binding to a sequence located 1 kb upstream of the gene's transcription start site. Finally, we provide evidence that Cbt nuclear localization is mediated by a monopartite NLS located at the N-terminal region of the protein (_71_PNKKPRL_77_), which is conserved in Cbt orthologs from other *Drosophila* species and insects. Genetic interaction assays and immunostaining using *importin-α* mutant strains suggested that the Importin-α2 protein is involved in Cbt nuclear import in *Drosophila*. Together, these results expand our understanding of the mechanisms of Cbt transcriptional regulation and nuclear import, which reveal the biochemical similarities and differences between vertebrate TIEG proteins and Cbt.

## Materials and Methods

### Plasmid constructs

pIE-β-GalCbt_1–428_, peGFP-Cbt_1–428_ and pIE-Gal4 constructs were generated by in-frame cloning of the *cbt* and *Gal4* coding regions, obtained by PCR amplification using the Pwo Polymerase (Roche diagnostics GmbH, Mannheim, Germany) and the oligos described in Table 1, into the pIE-β-Gal vector without β-Gal stop using the SalI- BamHI sites [43], the peGFP-C3 vector (Clontech Laboratories, Mountain View, CA) using the EcoRI-Asp718I sites and the pIE1-3 vector (Novagen, Madison, WI, USA) using the NotI-Asp718I sites, respectively. Other constructs used in this work were obtained by PCR amplification using pIE-β-GalCbt_1–428_ and peGFPCbt_1–428_ as templates and the oligos described in Table 1, followed by cloning into the pIE-β-Gal, peGFP-C3 and pIE-Gal4 vectors. All of the constructs were confirmed by DNA sequencing. The pG5DE5tkLuc plasmid, which contains the *luciferase* gene under the control of the UAS sequence, was a gift of Dr. Courey (UCLA, Los Angeles, CA, USA). Site-directed mutagenesis was carried out on the pIE-β-GalCbt_1–77_ construct to mutate lysines 73 and 74 (K73 and K74) to asparagine (N). Mutagenesis was performed by GenScript (NJ, USA).

**Table 1:** PCR-generated constructs and associated primers.

Construct	Oligonucleotides 5′-3′
pIE-β-GalCbt_1–428_	F (SalI):ACGCGTCGACGGGCGGCGGC**ATG**GACATGGATACCTTGC
	R (BamHI):CGCGGATCCCG**TCA**TCATCGCTGCAGTTGAAG
pIE-β-GalCbt_262–428_	F(SalI): GTCGACGCGGCCGCCCAGGCGGCGGCCA
	R (BamHI):CGCGGATCCCG**TCA**TCATCGCTGCAGTTGAAG
pIE-β-GalCbt_1–141_	F (SalI):ACGCGTCGACGGGCGGCGGC**ATG**GACATGGATACCTTGC
	R (BamHI):CGCGGATCCCG**TCA**GTTGACCCGCATGATGAC
pIE-β-GalCbt_1–108_	F (SalI):ACGCGTCGACGGGCGGCGGC**ATG**GACATGGATACCTTGC
	R (BamHI): CGGATCCCG**TCA**CAGTGGGTGGTACTGAAC
pIE-β-GalCbt_1–77_	F (SalI):ACGCGTCGACGGGCGGCGGC**ATG**GACATGGATACCTTGC
	R (BamHI): CGCGGATCCCG**TCA**CAAACGGGGTTTCTT
pIE-β-GalCbt_1–70_	F (SalI):ACGCGTCGACGGGCGGCGGC**ATG**GACATGGATACCTTGC
	R (BamHI): CGCGGATCCCG**TCA**AACCGCTATTTCGGGAGCCTCGTCTT
pIE-β-GalCbt_1–70KKPR_	F (SalI):ACGCGTCGACGGGCGGCGGC**ATG**GACATGGATACCTTGC
	R (BamHI):CGCGGATCCCG**TCA**ACGGGGTTTCTTAACCGCTATTCG
pIE-β-Gal-_PNKKPRL_	F (NotI): GCGGCCGC **ATG**AGCGAAAAATACATCG
	R (SalI):CGCGTCGAC **TCA**GTTTGCCCCAAAGAACAACCCTTTTTGACACCAGA
pIE-_PNKKPRL_-β-Gal	F (NotI): GCGGCCGC **ATG**CCCAACAAGAAACCCCGTTTGAGCGAAAAATACATCGTC
	R(BamHI): GGATCCTTATTACGTCGACCCTTTTGACACCAGACCAAC
pIE-β-GalCbt_37–77_	F (SalI):ACGGGTCGACGAAGGCAAAGCTCAAG
	R (Bam-HI):CGCGGATCCCG**TCA**CAAACGGGGTTTCTT
peGFPCbt_1–428_	F (EcoRI): CCGGAATTCCG**ATG**GACATGGATACCTTGC
	R (EcoRI):TATGAAT**TC** **A**TGGACATGGATACCTTGC
peGFPCbt _1–292_	F (EcoRI): CCGGAATTCCG**ATG**GACATGGATACCTTGC
	R (KpnI/Asp718I):CGGGGTACCC**TCA**GGGTCGCTCGCCGGTGTG
peGFPCbt_1–322_	F (EcoRI): CCGGAATTCCG**ATG**GACATGGATACCTTGC
	R (KpnI/Asp718I): CGGGTACCC**TCA**TTTCTTTTCACCGGTGTG
PeGFPCbt_1–262_	F (EcoRI): CCGGAATTCCG**ATG**GACATGGATACCTTGC
	R (KpnI/Asp718I):CGGGGTACCC**TCA**GATGCGACTTCTGGTGGC
PeGFPCbt_262–428_	F (KpnI/Asp718I):CCGGAATTCCGACGGCCGCCCAGGCGGCGGCCA
	R (EcoRI):TATGAAT**TC** **A**TGGACATGGATACCTTGC
pIE-Gal4_STOP_	F(NotI):AATGCGGCCGCAGAC**ATG**AAGCTACTGTCTTCT
	R (BamHI):CGCGGATCCGTACAG**TCA**ACTGTCTTTGACCTT
pIE-Gal4_ΔStop_	F(NotI):AATGCGGCCGCAGAC**ATG**AAGCTACTGTCTTCT
	R (BamHI):CGCGGATCCGTACAGACTGTCTTTGACCTT
pIE-Gal4Cbt_1–428_	F (BamHI): CGCGGATCCGC**ATG**GACATGGATACC
	R (BamHI):CGCGGATCCG**TCA**TCGCTGCACTTGAAGCAG
pIE-Gal4Cbt_1–262_	F(BamHI):CGCGGATCCGC**ATG**GACATGGATACC
	R (BamHI):CGCGGATCCG**TCA**GTAGATGCGACTTCTGGTGG
pIE-Gal4Cbt_1–182_	F (BamHI): CGCGGATCCGC**ATG**GACATGGATACC
	R (BamHI):CGCGGATCCG**TCA**TGGTGTCTCGGGTGTTT
pIE-Gal4Cbt_1–165_	F (BamHI): CGCGGATCCGC**ATG**GACATGGATACC
	R (BamHI):CGCGGATCCGAGGAGG**TCA**TCGCGAGTTCATTTTGAACTTGAGATT
pIE-Gal4Cbt_173–428_	F (BamHI):CGCGGATCCGAGGAGGTCGCGA**ATG**GCCGCCCAGGCGGCGG
	R (BamHI):CGCGGATCCG**TCA**TCGCTGCACTTGAAGCAG
pIE-Gal4Cbt_261–347_	F (BamHI):CGCGGATCCGC**ATG**ATCTACGAGTGCAGT
	R (BamHI): CGCGGATCCGC**TCA**GTCCTTGTTGTGCCG
pIE-Gal4Cbt_345–428_	F (BamHI):CGCGGATCCGC**ATG**AACAAGGACAAGGCG
	R (BamHI):CGCGGATCCG**TCA**TGGACATGGATACCTTGC
pIE-Gal4Cbt_261–389_	F (BamHI):CGCGGATCCGC**ATG**ATCTACGAGTGCAGT
	R (BamHI):CGCGGATCCGC**TCA**AGCGCTGGAGCCCGC

Sequences recognized by restriction enzymes (in parentheses) are underlined. The start and stop codons are in bold. F, forward primer; R, reverse primer.

#### Fly stocks

For genetic interaction assays, a *sev*-Gal4>UAS-*Cbt_FL_* line was generated via recombination. The following lines were obtained from the Bloomington Stock Center (http://flystocks.bio.indiana.edu/): y^1^ w^*^; P{EP}kapα1^G4113^, *y^1^ w^67c23^; P{EPgy2}pen^EY09095^*, *w^1118^; P{EP}kapα3C^G8397^/TM6C*, *Sb^1^* and *71B*-Gal4. The *w;impα2^D14^/Cyo Actin-GFP* stock [44] was a gift of Dr. Schwarz (Harvard Medical School, Boston, MA, USA). The following *importin* RNAi lines were obtained from the Vienna *Drosophila* RNAi Center (http://stockcenter.vdrc.at/control/main): *w^1118^;P {GD13960}v28920/Cyo*, *w^1118^;P {GD10665}v34265* and *w^1118^;P {GD14213}v36104*. All *Drosophila* strains were maintained at 25°C.

### Cell culture and transfection conditions

*Drosophila melanogaster* Schneider 2 cells (S2) and *Spodoptera exigua* cells (Sec301) were grown at 25°C in Schneider's *Drosophila* Medium with L-Glutamine (Biological Industries, Jerusalem, Israel/Invitrogen, Carlsbad, CA, USA) supplemented with 10% fetal bovine serum (FBS, Invitrogen) and 1% penicillin/streptomycin (Invitrogen). Chinese hamster ovary (CHO-K1) cells from *Cricetulus griseus* were grown at 37°C and 5% CO_2_ in DMEM/F-12 medium (Gibco/Invitrogen) supplemented with 10% FBS and 1% penicillin/streptomycin. For subcellular localization assays, 1×10^6^ cells/ml were seeded onto coverslips in 24-well plates. After 24 h, cells were transfected with 0.5–1 µg of DNA for 5 h using 8 µl of Cellfectine reagent (Invitrogen) for insect cells and 6 µl of FugeneHD (Roche) for CHO-K1 cells.

### Repression and UAS/Gal4-based transcriptional assays

For the repression assay, S2 cells were transfected with 0.5 µg of the pMT-Cbt-V5 plasmid, 0.5 µg of the pHStinger-Prom1-2 plasmid (see [13]) and 0.1 µg of the PAC-cherry vector (to normalize for transfection efficiency) in 6-well plates at 70–90% confluence according to supplier's recommendations (Transfection Custom Insect Reagent, Mirus Bio Corp, Madison, WI). Expression of the recombinant protein was induced by incubation in medium containing 0.25/0.75 µM copper sulfate for 48 h. Fluorescence was measured using a TECAN infinit® 200 plate reader. For the UAS/Gal4 assays, S2 cells were co-transfected in 24-well plates with 0.075 µg of each construct, 0.1 µg of the pG5DE5tkLuc vector and 0.1 µg of the PAC-Renilla vector to normalize for transfection efficiency. The transfection protocol was the same as for the repression assay. After 48 h, cells were lysed and incubated with the Dual Luciferase Assay Kit (Promega, Madison, WI, USA) following the manufacturer's instructions. Luciferase activity was measured using a Modulus single tube multimode reader.

### Immunochemistry and scanning electron microscopy

Cells were washed with phosphate-buffer saline (PBS) 16–24 h after transfection, fixed in freshly prepared 4% paraformaldehyde/PBS for 20 min, permeabilized with 0.1% Triton-X-100/PBS for 5 min, and blocked with 1% BSA/PBS for 30 min. After incubation with rabbit anti-β-Gal (1∶1000, Cappel/ICN Biomedicals, Ohio, USA) and mouse monoclonal anti-Lamin (1∶200) (Developmental Studies Hybridoma Bank, Iowa, USA) antibodies for 2 h, cells were washed three times with 0.5% BSA/PBS, incubated with anti-rabbit-FITC (1∶200, Calbiochem EMD Biosciences Inc., La Jolla, CA) and anti-mouse-Cy3 (1∶200, Invitrogen) antibodies for 1 h, washed three times with PBS, mounted in Vectashield (Vector Laboratory, Peterborough, UK) and examined under a Leica TCS SP1 confocal microscope. Ovaries were dissected as described [45] and stained using the anti-CbtΔZn antibody [13]. Scanning electron microscopy analysis of adult eyes was performed following the critical point dry method using a Hitachi S4100 microscope, as previously described [46].

### Western blotting

Cells were collected 16–24 h after transfection, washed with PBS and scraped into 50 µl of sodium dodecyl sulfate-polyacrylamide gel electrophoresis (SDS-PAGE) loading buffer. After boiling, aliquots were subjected to 10% SDS-PAGE. Subsequently, proteins were electrotransferred to PDVF membranes (Roche) and detected with rabbit anti-β-Gal (1∶5000, Cappel) or anti-eGFP (1∶2000, Roche) primary antibodies. The secondary antibody was peroxidase-labeled anti-rabbit (1∶3000, Calbiochem). The ECL western blotting detecting reagent (Pierce, Rockford, IL, USA) was then used to reveal the chemiluminescence.

### Computational analyses

PSORT II (http://psort.nibb.ac.jp/form2.html) and cNLS Mapper (http://nls-mapper.iab.keio.ac.jp/cgi-bin/NLS_Mapper_form.cgi) software programs were used to predict NLSs and nuclear export signal (NESs). Multiple alignments of protein sequences were performed with the ClustalW algorithm [47]. The automated protein structure homology-modeling server SWISS-MODEL (http://swissmodel.expasy.org/) was used to analyze secondary structure. NetPhos (http://www.cbs.dtu.dk/services/NetPhos/) and NetPhosK (http://www.cbs.dtu.dk/services/NetPhosK/) programs were used to predict putative phosphorylation sites and specific kinase-binding sites. The GENPEPT database (invertebrate and vertebrate sections; GenBank) was searched with the PX(K/R)KX(R/L) string (where X = any or no amino acid) using the Scansite program (http://scansite.mit.edu; Quick matrix method).

## Results

### Cabut acts as a potent transcriptional repressor and regulates its own transcription

**Figure 1:**
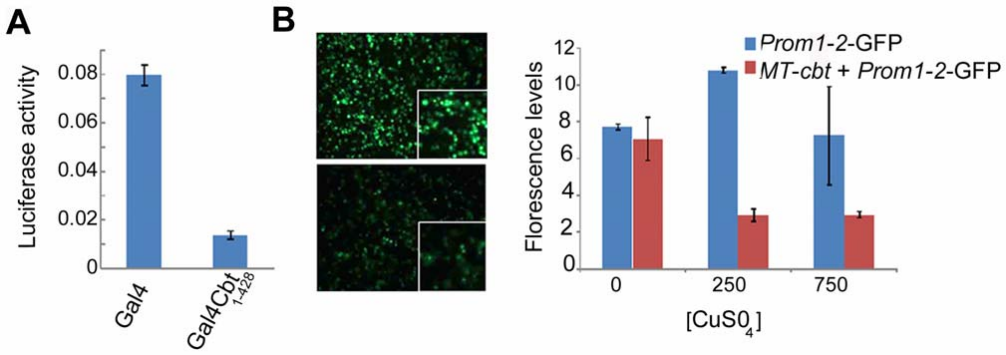
Cabut functions as a transcriptional repressor in S2 cells and regulates its own transcription. (A) Expression of Gal4Cbt_1–428_ led to repression of luciferase activity levels relative to a control Gal4 protein. Luciferase activity was measured 48 h after transfection. pRENILLA was used to normalize for cell number, transfection efficiency, and general effects on transcription (luciferase activity = firefly luciferase/renilla luciferase). (B) S2 cells transfected with *Prom1-2*-GFP as a control or co-transfected with *Prom1-2*-GFP and MT-*cbt*. The MT promoter was induced by exposing the cells to medium containing copper (upper picture, no copper; lower picture, plus copper). Fluorescence levels were reduced following transcriptional induction of Cbt by copper in cells co-transfected with *Prom1-2*-GPF and MT-*cbt* (left panels). Fluorescence was measured 48 h after induction (right panel). pCHERRY was used for normalization (Fluorescence levels = GFP/CHERRY). In A and B, data are presented as the mean ± SD of three replicates.

The UAS/Gal4 fusion system [48] was used in *Drosophila* S2 cells to determine the transcriptional repression and/or activation activity of Cbt. Gal4 fusion proteins have little transcriptional background interference in *Drosophila* cells because of their yeast origin. For these experiments, we generated the pIE-Gal4Cbt_1–428_ construct, in which the full-length Cbt protein was fused to the Gal4 DBD. This construct was cotransfected into S2 cells with a reporter construct containing Gal4-binding sites upstream of the firefly *luciferase* gene (the UAS-Luciferase vector). A significant repression of *luciferase* transcription (∼6-fold) was observed in this experiment as compared to transfection with the Gal4 DBD alone (Figure 1A). This result indicates that the Cbt full-length protein is able to repress transcription. Interestingly, preliminary results obtained in chromatin immunoprecipitation assays combined with genomic microarrays (ChIP-on-chip) in Canton-S embryos during dorsal closure (Y.B. and N.P. in collaboration with the modENCODE Project, unpublished results) suggested that Cbt was able to bind to a GC-rich genomic region 1 kb upstream of the *cbt* transcriptional start site (2 L: 479789,480740, Figure S1). Previous electrophoretic mobility shift assays (EMSAs) performed using the Cbt DBD region have demonstrated Cbt binding to GC-rich regions [49]. To confirm this result, we co-transfected *Drosophila* S2 cells with a construct in which the expression of a Cbt-V5 fusion protein was controlled by the methalotionein promoter (*MT*-Cbt) and a plasmid containing that region (named as Prom1-2 in [13]) fused to GFP (*Prom1-2*-GFP). To induce Cbt-V5 expression, the transfected cells were grown in Cu-supplemented medium. Indeed, induction of Cbt expression led to a significant reduction in GFP levels (Figure 1B). Taken together, these results show that Cbt acts as a transcriptional repressor, similar to the mammalian TIEG proteins, at least in S2 cells. Moreover, these experiments also demonstrate that the *cbt* gene autoregulates its own expression via a negative autoregulatory feedback mechanism, as has been shown for several genes involved in circadian rhythms [50], [51], [52]. However, this mechanism has not been previously reported for vertebrate TIEG proteins.

### Identification of transcriptional repressor domains in the Cbt protein

**Figure 2:**
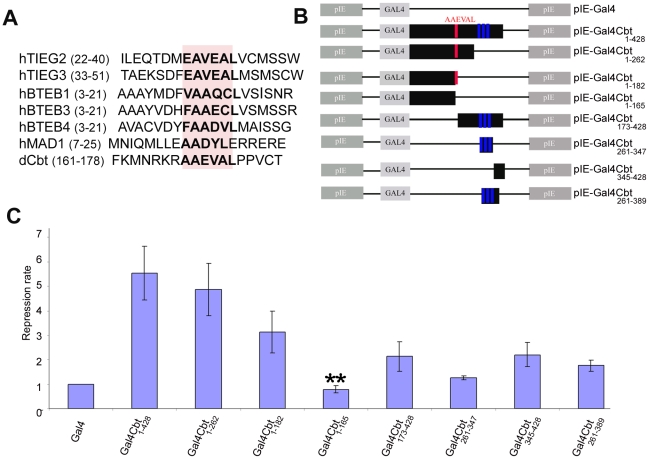
Identification of domains required for Cabut's transcriptional repressor activity. (A) Multiple alignment of the Sin3A interacting domains (SID) from *Drosophila* Cbt and several vertebrate TFs such as the MAD protein and mebers of the Sp1 family (TIEG, BTEB). Note that this domain is highly conserved in sequence (marked by a pink rectangle and residues in bold) but not with respect to location within the protein. (B) Schematic representation of the CbtGal4 fusion constructs transfected into S2 cells to identify transcriptional regulatory domains in the Cbt protein. The AAEVAL sequence is indicated in red and the C_2_H_2_ zinc fingers in blue. (C) Degree of *luciferase* repression obtained in UAS/Gal4 assay in S2 cells transiently transfected with the constructs shown in (B). Repression rate = luciferase activity of pIE-Gal4/luciferase activity of tested construct. pRENILLA was used for normalization as in Fig. 1. Data are presented as the mean ± SE (n≥5). Note that removal of the AAEVAL sequence completely abolishes the repressor activity of the Cbt protein (asterisks indicate p-value<0.01, t-Student's test).

Previous studies showed that the repressor activity of the human TIEG1, TIEG2 and murine TIEG3 proteins in CHO-K1 and OLI-neu cells is mediated by a Sin3A-interacting domain (SID) (with an EAVEAL consensus sequence), which is required for its interaction with the Sin3A co-repressor and is located at the N-terminal region of the proteins within an α-helix motif [31], [32]. Site-mutagenesis analyses revealed that the first alanine (A) residue and the α-helical structure are important for the recognition of this domain by the Sin3A co-repressor [53]. Because Cbt is able to repress transcription in S2 cells, we decided to determine whether the SID is conserved in the *Drosophila* protein. Multiple alignments of Cbt and several vertebrate TFs such as MAD and members of the Sp1 family (TIEG, BTEB), revealed that a similar domain, with equivalent residues, is present in Cbt but in a different region of the protein (_168_AAEVAL_173_ in Figure 2A). Secondary structure analysis of the Cbt protein using the SWISS-MODEL server [54] confirmed the conservation of an α-helix motif in the Cbt AAEVAL sequence (Figure S2). The presence of this domain could explain the transcriptional repressor activity of Cbt in S2 cells described above. Interestingly, this sequence is also conserved in all *Drosophila* species analyzed (Figure S2), suggesting that Cbt orthologs in these species may present a similar transcriptional activity. To determine whether this sequence is responsible for Cbt's repressor activity and to identify other possible transcriptional repression and/or activation domains in the protein, we generated a collection of Cbt Gal4 DBD fusion proteins (Figure 2B). Plasmids expressing these fusion proteins were cotransfected with the UAS-Luciferase vector (as described in Materials and Methods) into S2 cells, which endogenously express the dSin3A co-repressor [55]. Our results show that fusion proteins containing the AAEVAL sequence (pIE-Gal4Cbt_1–428_, pIE-Gal4Cbt_1–262_, pIE-Gal4Cbt_1–182_) strongly repressed *luciferase* expression (up to 5-fold) in transfected cells (Figure 2C). This repressive effect is completely dependent on that sequence, as cotransfection with the pIE-Gal4Cbt_1–165_ construct fails to repress the UAS-Luciferase reporter (Figure 2C). Thus, this result indicates that the AAEVAL sequence is essential for Cbt-mediated repression in S2 cells. In addition, we found that the C-terminal region of the Cbt protein, which does not contain the SID (pIE-Gal4_173–428_), was able to reduce luciferase expression by ∼1.5-fold (Figure 2C), thereby indicating that this region of the protein may contain one or more repression domains close to the DBD. Multiple alignments of the *Drosophila* Cbt orthologs revealed the presence of two regions in the Cbt C terminus that were highly conserved in all proteins analyzed (C4 and C5 in Figure S2). To confirm this possibility and test the transcriptional activity of both the DBD and the C4 and C5 conserved sequences, different constructs were generated in which truncated Cbt proteins containing only the Cbt DBD (pIE-Gal4Cbt_261–347_), the Cbt C-terminal region including the C4 and C5 sequences (pIE-Gal4Cbt_345–428_), and the Cbt DBD plus the C4 sequence (pIE-Gal4Cbt_261–389_) were fused to the Gal4 DBD (Figure 2B) and cotransfected with the UAS-Luciferase vector into *Drosophila* S2 cells. Our results show that the DBD of Cbt by itself is not able to activate *luciferase* expression (Figure 2C). In addition, we found that the C-terminal region of Cbt without the zinc finger domains (compare pIE-Gal4 to Gal4Cbt_345–428_) can significantly repress *luciferase* expression (∼1.5-fold) (Figure 2C). Thus, our results show that the conserved sequence _379_LRAIAPA_385_, which we have called REP1/C4 (Figure 2D and Figure S2) and which is not present in the TIEG proteins, appears to have a mild repressive effect on *luciferase* expression (Figure 2C) in the UAS/Gal4 assays and could be in part responsible for the transcriptional repressor activity of the Cbt protein.

### The Cys_2_His_2_-zinc finger domains of Drosophila Cabut are not essential for its nuclear localization in S2 cells but function as an NLS in mammalian CHO-K1 cells

**Figure 3:**
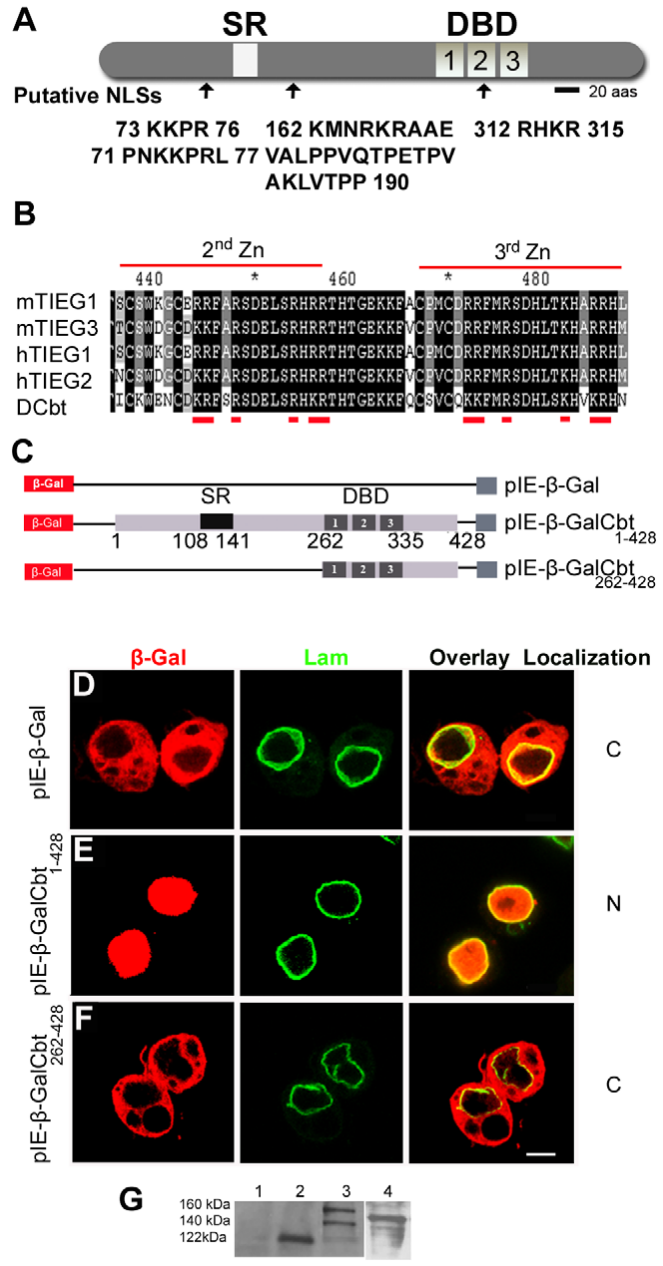
The Cabut zinc finger region is not essential for nuclear localization in S2 cells. (A) Schematic representation of the *Drosophila* Cbt protein, in which the locations of the Ser-rich (SR) region and the DBD are indicated. Sequences, coordinates and locations (arrows) of the predicted NLSs are also indicated. (B) Multiple alignment of the second and third zinc fingers of murine TIEG1 and TIEG3, human TIEG1 and TIEG2 and *Drosophila* Cbt. Red lines below the alignment indicate the amino acids included in the murine and human TIEG NLSs. (C) Schematic representation of the β-GalCbt fusion constructs used to transfect S2 cells. The locations of the SR region and the DBD are indicated by boxes. (D–F) Localization of β-Gal fusion proteins in S2 cells transiently transfected with the constructs shown in (C). Cells were stained with anti-β-Gal (red; first panel) and anti-Lam (green; second panel) to mark nuclear membranes. The overlay panel depicts double staining of cells with both antibodies, and the localization of the fusion proteins is shown in the fourth panel (C, cytoplasmic; N, nuclear). Wild-type β-Gal was located in the cytoplasm (D), but the β-GalCbt fusion protein translocated to the nucleus (E). However, the Cbt zinc finger region alone was not able to translocate β-Gal to the nucleus (F). Scale bar: 10 µm. (G) Western blot of protein extracts from S2 cells transfected with the constructs shown in (C) and stained with anti-β-Gal. (1) Non-transfected cells, (2) empty pIE-β-Gal vector (∼122 kDa), (3) pIE-β-GalCbt_1–428_ (∼160 kDa) and (4) pIE-β-GalCbt_262–428_ (∼140 kDa). Cells transfected with the pIE-β-GalCbt_1–428_ construct presented some degradation that did not affect the subcellular localization of the fusion protein.

**Figure 4:**
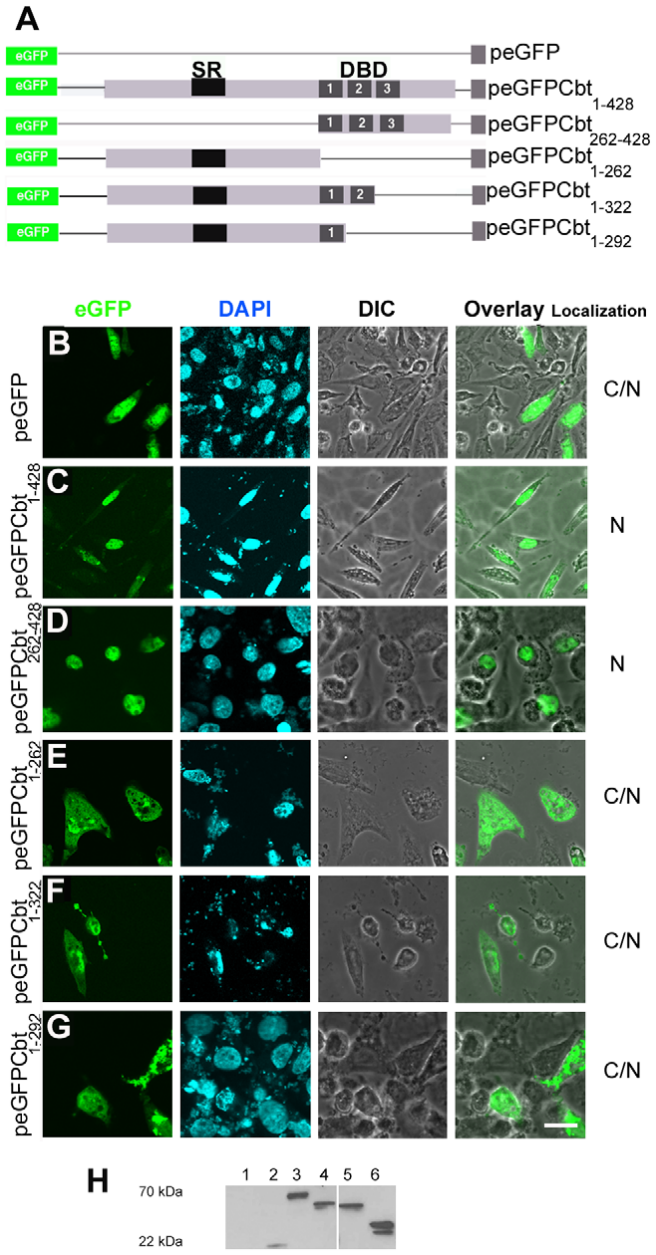
The Cabut zinc finger region is not essential for nuclear localization in CHO-K1 cells. (A) Schematic representation of the eGFPCbt fusion constructs used to transfect CHO-K1 cells. The locations of the SR region (black rectangle) and the zinc fingers (1, 2, 3, grey rectangles) are indicated. (B–G) Localization of eGFP fusion proteins in CHO-K1 cells transiently transfected with the constructs shown in (A). Cells were immunostained with anti-eGFP (green, first panel) and DAPI (blue; second panel). Differential interference contrast (DIC) was used to visualize cell boundaries (third panel). The overlay panel shows DIC and anti-eGFP staining. The localization of the fusion proteins is shown in the fourth panel (C, cytoplasmic; N, nuclear). Wild-type eGFP was located in the cytoplasm and the nucleus (B), but the eGFPCbt fusion protein translocates to the nucleus (C). With the exception of the peGFPCbt_262–428_ fusion protein, which lacks the N-terminal region of the Cbt protein (G), all Cbt deletions affecting the zinc finger region showed cytoplasmic localization (D–F). Scale bar: 10 µm. (H) Western blots of protein extracts from CHO-K1 cells transfected with the constructs shown in (A) and stained with anti-GFP. (1) Non-transfected cells, (2) empty peGFP vector (∼27 kDa), (3) peGFPCbt_1–428_ (∼70 kDa), (4) peGFPCbt_1–322_ (∼60 kDa), (5) peGFPCbt_1–292_ (∼60 kDa) and (6) peGFPCbt_1–262_ (∼50 kDa).

Proteins larger than 45 kDa in size are unable to pass the nuclear membrane by passive diffusion [56]. Because we recently showed that Cbt is a nuclear protein with a theoretical molecular weight of 48 kDa [13], we assumed that it contains at least one functional NLS, although it could also be transported to the nucleus via an interaction with another NLS-containing protein. Indeed, four potential NLSs were identified in the Cbt protein using PSORT II [57] and cNLSMapper [58] programs (Figure 3A). Two of these putative NLSs were _73_KKPR_76_ and _71_PNKKPRL_77_, which were predicted by both programs and located in the N-terminal region. The third sequence was a bipartite NLS, _162_KMNRKRAAEVALPPVQTPETPVAKLVTPP_190_, which yielded the highest score in the cNLSMapper program. The fourth sequence was _312_RHKR_315_, which is located within the second Cbt zinc finger domain and predicted by both programs. A similar sequence (RHRR) was also found in the second zinc finger domain of the murine TIEG3 protein and is included within a functional bipartite NLS that is essential for TIEG3 nuclear import (Figure 3B) [19], [32]. Because the zinc finger domains are highly conserved between Cbt and the TIEG proteins (Figure 3B and [14]), we decided to test the role of this putative NLS in Cbt nuclear localization. For doing so, we transiently transfected *Drosophila* S2 cells with the pIE-β-GalCbt_262–428_ construct, in which the C-terminal region of Cbt containing the zinc finger region was fused to the *E. coli* β-Galactosidase (β-Gal, 116 kDa) cytoplasmic protein (Figure 3C, D). The pIE-β-GalCbt_1–428_ construct, in which β-Gal is fused in frame to the full-length Cbt protein (Figure 3C), was used as a control. In these experiments, anti-Lamin (Lam) immunostaining was used to define the nuclear region. Double staining of the transfected cells with anti-β-Gal and anti-Lam antibodies showed that while the β-GalCbt_1–428_ protein was exclusively localized in the nucleus (Figure 3E), β-GalCbt_262–428_ was completely excluded from this cellular compartment, remaining in the cytoplasm (Figure 3F). Western blot analyses of transfected cell extracts were performed to confirm the integrity of the fusion proteins (Figure 3G). These assays showed that while the full-length Cbt protein appears to be partially degraded in S2 cells extracts (without affecting the NLS), the β-GalCbt_262–428_ protein appears to be stable (Figure 3G). This result demonstrates that the putative NLS identified in the Cbt zinc finger region does not play a role in the protein's nuclear localization and suggests that the Cbt NLS is probably located at its N terminus. However, due to the high similarity between the Cbt and TIEG zinc finger domains, where essential basic amino acids are conserved (Figure 3B and [32]), we tested whether this region of the Cbt protein could function as an NLS in mammalian cells. Several expression constructs were generated: truncated Cbt proteins lacking the N-terminal region of Cbt (peGFPCbt_262–428_), the complete zinc finger region (peGFPCbt_1–262_), the third zinc finger (peGFFCbt_1–322_), and both the second and third zinc fingers (peGFPCbt_1–292_) were fused to eGFP (Figure 4A) and used to transiently transfect mammalian CHO-K1 cells. We found that the eGFP protein alone localizes to both the cytoplasm and the nucleus by passive diffusion due to its molecular weight (30 kDa) (Figure 4B). Cells transfected with the peGFPCbt_1–428_ or peGFPCbt_262–428_ construct showed exclusively nuclear eGFP localization (Figure 4C, D). However, cells transfected with deletion constructs affecting the zinc finger domains presented both cytoplasmic and nuclear eGFP signals (Figure 4E–G).Western blot analysis of cell extracts showed no degradation of the fusion proteins (Figure 4H). These results are in agreement with those obtained for the TIEG3 protein in HeLa and OLI-neu cells [32] and indicate that the Cbt bipartite NLS within the second and third zinc fingers is functional in mammalian cells, suggesting that different nuclear import mechanisms for this protein are being used in *Drosophila* and mammalian cells.

### The PNKKPRL motif is necessary for Cbt nuclear localization

**Figure 5:**
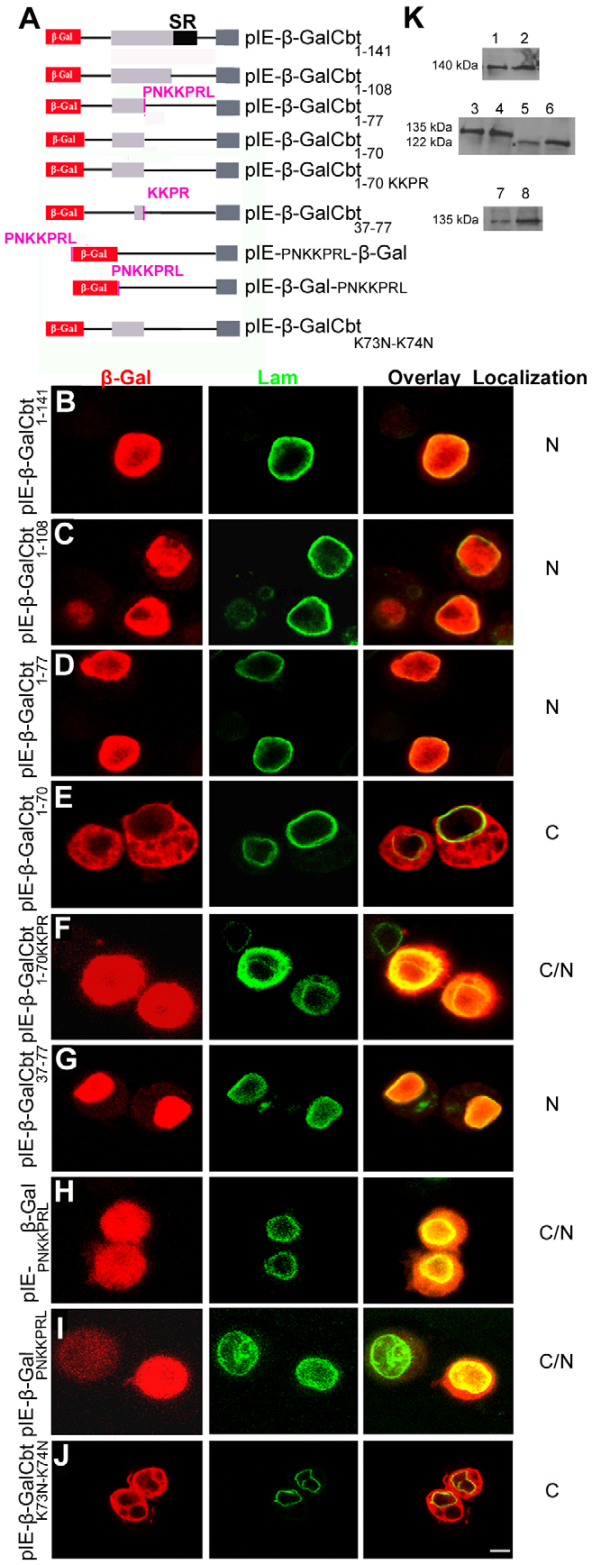
The _71_PNKKPRL_77_ sequence is the NLS of the Cabut protein. (A) Schematic representation of the β-GalCbt fusion constructs transfected into S2 cells to determine whether the _162_KMNRKRAAEVALPPVQTPETPVAKLVTPP_190_ and/or _71_PNKKPRL_77_ sequences are functional NLSs. The Serine-rich (SR) domain is shown in black and the PNKKPRL sequence in fuchsia. (B–J) Localization of β-Gal fusion proteins in S2 cells transiently transfected with the constructs shown in (A). Cells were stained with anti-β-Gal (red; first panel) and anti-Lam (green; second panel) to mark nuclear membranes. The overlay panel depicts double staining of cells with both antibodies, and the localization of the fusion proteins is shown in the fourth panel (C, cytoplasmic; N, nuclear). Note that fusion proteins lacking or containing a mutated _71_PNKKPRL_77_ sequence (E and J) were cytoplasmic. The _71_PNKKPRL_77_ sequence was able to translocate β-Gal to the nucleus when fused to either the N- or the C-terminus of the protein (H and I). Scale bar: 10 µm. (K) Western blots of protein extracts from S2 cells transfected with the constructs shown in (A) and stained with anti-β-Gal. (1) pIE-β-GalCbt_1–141_ (∼140 kDa), (2) pIE-β-GalCbt_1–108_ (∼140 kDa), (3) and (7) pIE-β-GalCbt_1–77_ (∼135 kDa), (4) pIE-β-GalCbt_1–70_ (∼135 kDa), (5) pIE-_PNKKPRL_-β-Gal (∼120 kDa), (6) pIE-β-Gal-_PNKKPRL_ (∼120 kDa) and (8) pIE-β-Gal-Cbt_K73N–K74N_ (∼135 kDa).

Our results indicate that a functional NLS is located within the N-terminal region of the Cbt protein. To test whether any of the predicted sequences in that region are required for Cbt nuclear localization (Figure 3A), we transiently transfected S2 cells with several deletion constructs. First, we generated pIE-βGalCbt_1–141_ and pIE-βGalCbt_1–108_ constructs, encoding β-Gal fused to truncated Cbt proteins lacking either the bipartite NLS, _162_KMNRKRAAEVALPPVQTPETPVAKLVTPP_190_, or that sequence plus the SR domain (Figure 5A). Our results showed that both β-GalCbt fusion proteins were able to translocate to the nucleus (Figure 5B–C), thus indicating that neither the predicted bipartite NLS nor the SR region plays any role in Cbt nuclear localization and that an active NLS sequence is still retained in the truncated proteins. We next wanted to determine whether the overlapping _73_KKPR_76_ and _71_PNKKPRL_77_ sequences could act as functional NLSs. To do so, we generated constructs in which β-Gal was fused to Cbt N termini with (pIE-β-GalCbt_1–77_) or without these sequences (pIE-β-GalCbt_1–70_) (Figure 5A) and used them to transfect S2 cells. Immunostaining revealed that the removal of both sequences completely abolished the nuclear transport of the fusion proteins (Figure 5D, E), thus indicating that a functional NLS is present in this region. We next wanted to determine whether the KKPR residues, which are included within the PNKKPRL sequence and predicted as an NLS by the PSORT II program, were sufficient to target Cbt to the nucleus. Therefore, we generated the pIE-β-GalCbt_1–70KKPR_ construct (Figure 5A), in which only the KKPR sequence is present. Immunostaining of transfected S2 cells showed that the fusion protein was localized in both the nucleus and the cytoplasm (Figure 5F). Because the integrity of the protein was confirmed by western blot analysis (Figure 5K), this observation indicates that the nuclear transport of β-GalCbt_1–70KKPR_ is not efficient and suggests that the additional residues in the long NLS are necessary to increase the efficiency/rate of nuclear transport (compare Figure 5D to Figure 5F). We next wanted to determine whether the PNKKPRL sequence was sufficient to translocate a reporter protein to the nucleus. Therefore, we generated the pIE-β-Gal-_PNKKPRL_ and pIE-_PNKKPRL_-β-Gal constructs, in which the PNKKPRL sequence was fused in frame to either the C- or the N-terminal region of the β-Gal protein (Figure 5A). Immunostaining of transfected S2 cells revealed that the β-Gal protein was transported to the nucleus in both cases (Figure 5H–I), indicating that the PNKKPRL sequence is sufficient for nuclear import. Interestingly, this sequence is very similar to the SV40 large T antigen NLS (PKKKRKV) [59]. To determine which residues within the PNKKPRL sequence are important for Cbt nuclear transport, we performed site-directed mutagenesis of the basic lysine 73 and lysine 74 (K73 and K74) residues to asparagine (N) in the pIE-β-GalCbt_1–76_ construct (designated pIE-β-GalCbt_K73N–K74N_ in Figure 5A). Immunostaining of transfected S2 cells revealed that mutation of both K residues abolished Cbt nuclear transport (Figure 5J), indicating that they are essential for this process. Nuclear transport of the β-Gal protein fused to the PNKKPRL sequence was not perfectly efficient, as some of the protein remained in the cytoplasm. It is therefore likely that either additional regions of the Cbt protein or other factors must be involved in its nuclear transport. Because no other NLSs in the Cbt protein were predicted by the utilized bioinformatic programs, it is possible that other sequences in the N-terminal region of the protein could be required together with the NLS for Cbt nuclear import. Multiple alignments of the Cbt *Drosophila* orthologs allowed us to identify three highly conserved regions in that part of the protein (C1, C2 and C3 in Figure S2). To determine whether these regions could act cooperatively with the PNKKPRL sequence in Cbt nuclear import, we generated a construct in which the β-Gal protein was fused to a Cbt fragment encompassing residues 37–77 (pIE- β-GalCbt_37–77_) (Figure 5A), which lacked C1 and part of C2 and only contained C3 and the NLS. Immunostaining of transfected S2 cells revealed exclusive nuclear localization of the fusion protein, suggesting that the C3 region, but not the C1 or C2 region, may be required for Cbt nuclear translocation and could act cooperatively with the NLS (Figure 5G). It has been shown that post-translational modifications involving phosphorylation/dephosphorylation of NLSs and adjacent residues represent one of the mechanisms used to regulate nuclear import kinetics (reviewed in [60], [61]). It is therefore possible that Cbt nuclear import could be regulated by desphosphorylation/phosphorylation events (see Discussion).

Taken together, these results demonstrate that the PNKKPRL sequence in the Cbt protein is a functional NLS motif in which the central K residues are essential for nuclear transport and the P, N, R and leucine (L) residues are probably required to increase efficiency. We also speculate that the C3 region located upstream of the NLS might regulate this process and thus confirm previous observations regarding the regulatory roles of sequences flanking classical NLSs.

### The PNKKPRL NLS is conserved and functional in Cbt insect orthologs

**Figure 6:**
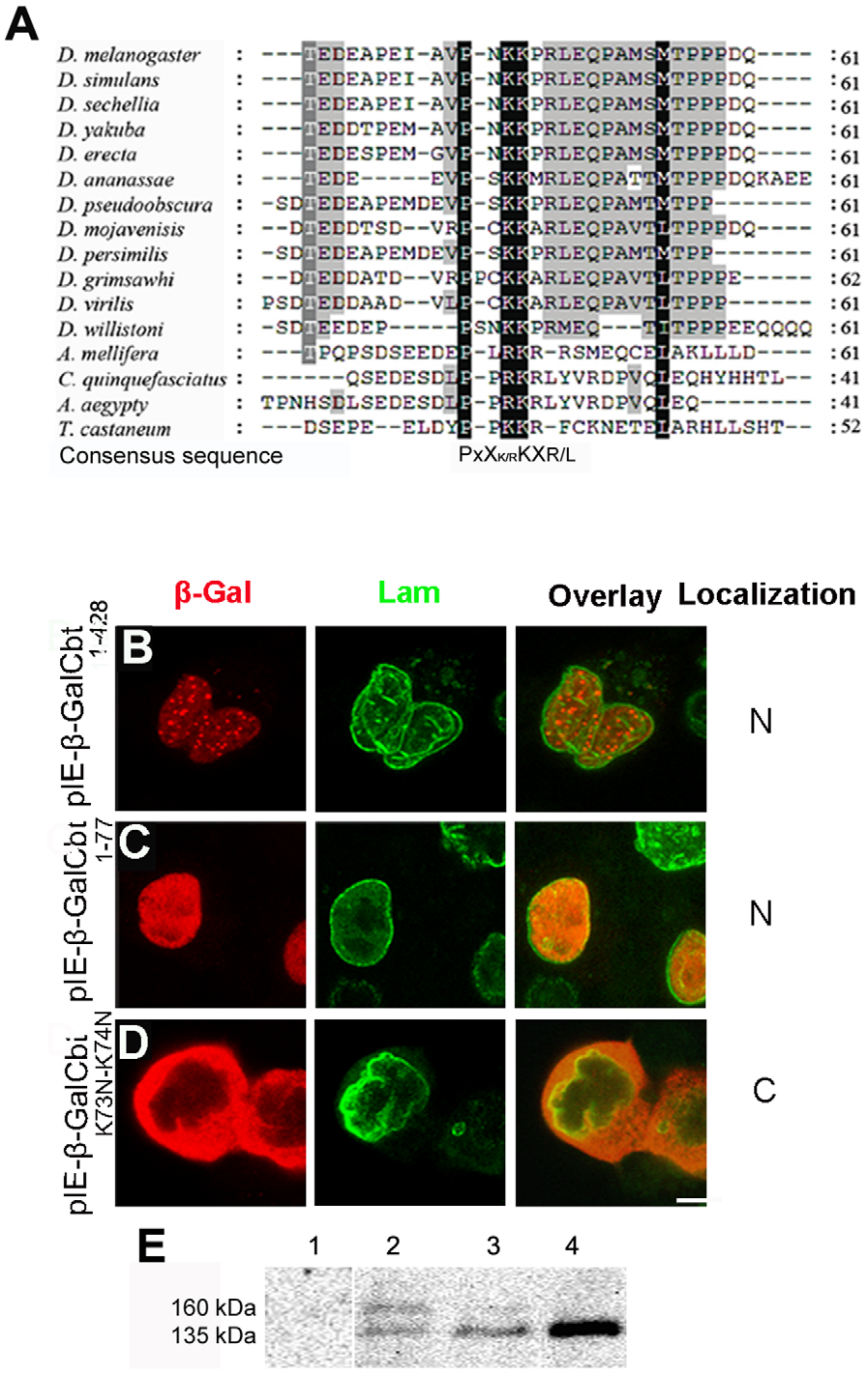
The PNKKPRL sequence is conserved and functional in insect Cabut orthologs. (A) Multiple alignment of Cbt proteins from *Drosophila* species and other insects, showing that the PNKKPRL motif is highly conserved. A consensus sequence for the putative NLS is shown below (X = any amino acid). (B–D) Localization of β-Gal fusion proteins in *Spodoptera exigua* Sec301 cells transiently transfected with the constructs shown in (A). Cells were stained with anti-β-Gal (red; first panel) and anti-Lam (green; second panel) to mark nuclear membranes. The overlay panel depicts double staining of cells with both antibodies, and the localization of the fusion proteins is shown in the fourth panel (C, cytoplasmic; N, nuclear). Note that all β-Gal fusion proteins containing the wild-type PNKKPRL sequence were translocated to the nucleus. Scale bar: 10 µm. (E) Western blots of protein extracts from Sec301 cells transfected with constructs shown in (A) and stained with anti-β-Gal. (1) Non-transfected Sec301 cells, (2) pIE-β-Gal-Cbt_1–428_ (∼160 kDa), (3) pIE-β-Gal-Cbt_1–77_ (∼135 kDa) and (4) pIE-β-Gal-Cbt_1–K73N–K74N_ (∼135 kDa).

To determine whether the Cbt NLS and other residues important for its nuclear import are conserved in other *Drosophila* species and insects, we performed multiple alignments of the amino acid sequences of their Cbt orthologs. Our results showed that the PNKKPRL sequence was conserved in the twelve *Drosophila* species analyzed (Figure 6A and Figure S2). Consistent with this, we have recently demonstrated the nuclear localization of Cbt proteins in several *Drosophila* species [13]. Moreover, P and basic residues in the PNKKPRL sequence were also found in the Cbt proteins of several other insects, including *Apis mellifera*, *Culex quinquefasciatus*, *Aedes aegyti* and *Tribolium castaneum*, although they present a divergent N-terminal region (Figure 6A). Similar analyses in other Cbt orthologs of *Ciona intestinalis*, *Strongylocentrotus purpuratus*, *Daphnia pulex* and vertebrate TIEGs revealed that the PNKKPRL sequence is not present in these proteins (data not shown). Thus, the consensus NLS of insect Cbt orthologs may be PX(K/R)KX(R/L) (X = any residue). To test whether the Cbt NLS is functional in other insects, we used the pIE-β-GalCbt_1–428_, pIE-β-GalCbt_1–77_ and pIE-β-GalCbt_K73N–K74N_ constructs (Figure 5A) to transiently transfect Sec301 cells from the beet armyworm *Spodoptera exigua*. Immunostaining revealed that both the full-length Cbt protein and a truncated form containing the PNKKPRL NLS but lacking the zinc finger domains and the predicted bipartite NLS were able to translocate the β-GalCbt fusion protein to the nucleus in *S. exigua* cells (Figure 6B–C). However, when the construct encoding β-Gal fused to a Cbt N-terminal region (1–77) with a mutated PNKKPRL sequence (pIE- β-GalCbt_K73N–K74N_) was transfected, the fusion protein was exclusively localized in the cytoplasm, as in *Drosophila* S2 cells (compare Figure 6D to Figure 5J). Western blot analyses confirmed that the proteins expressed in the transfected cells were of the correct size (Figure 6E). Taken together, these results indicate that the PX(K/R)KX(R/L) consensus motif is evolutionarily conserved and probably functional in insect Cbt orthologs. Its absence from TIEG proteins suggests that it evolved after the divergence between vertebrates and invertebrates.

Further searches in the GENPEPT protein database using the SCANSITE software [62] revealed a total of 57 proteins containing the PX(K/R)KX(R/L) sequence, 32 from invertebrates and 25 from vertebrates (data not shown). Among the identified invertebrate proteins, 53% were nuclear, including the *Drosophila* C_2_H_2_-zinc finger TFs Krüppel, Snail, Escargot and Scratch and the *A. gambiae* reverse transcriptase protein (Q868R2), suggesting that this sequence could be involved in the nuclear localization of other proteins, as predicted by the PSORT II program. Interestingly, the PX(K/R)KX(R/L) sequence is conserved and functional in the human BRCA1 (BReast CAncer Type 1) protein, a tumor suppressor protein involved in damaged DNA repair [63]. A _606_PKKNRLRRKS_615_ sequence that is similar to the Cbt NLS is involved in BRCA1 nuclear transport and interacts with the hSRP1α/importin-α2 protein [64].

### Importin-α2 is involved in Cbt nuclear import in ovaries

**Figure 7:**
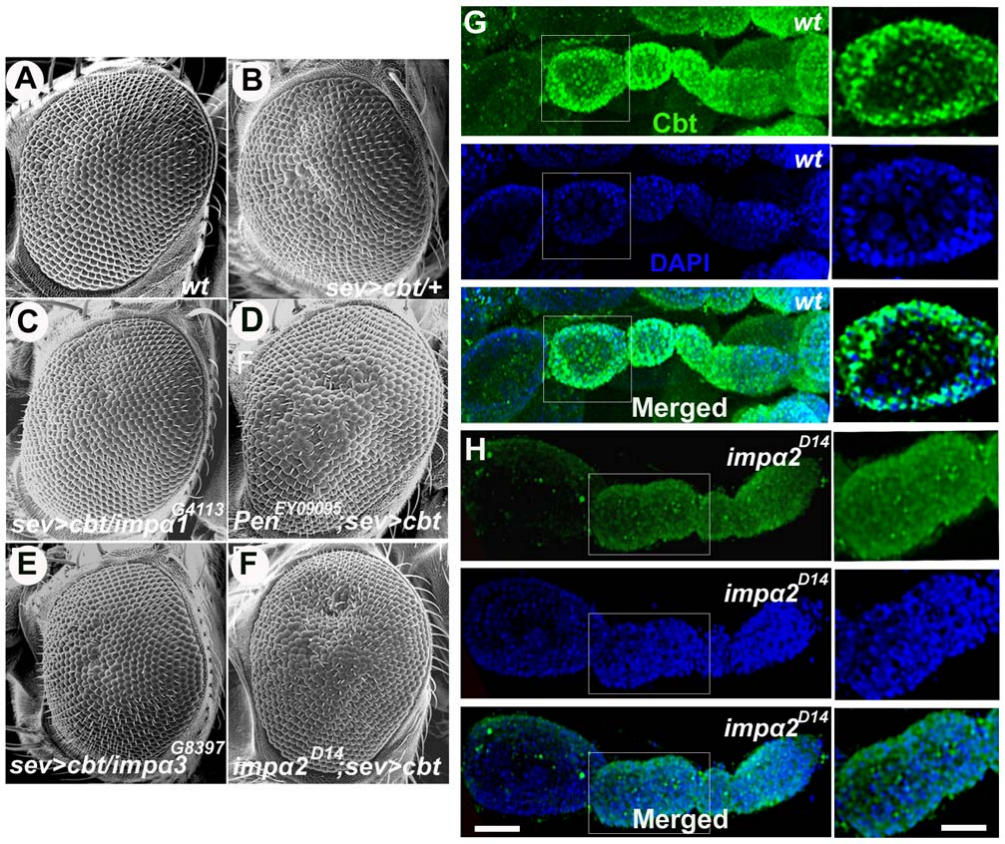
Importin-α2 is required for Cabut nuclear import in the *Drosophila* ovary. (A–F) Scanning electron micrographs of adult eyes of the indicated genotypes. Note that eye roughness in Cbt-overexpressing flies was dominantly enhanced when the *impα2* dosage was reduced (compare B to D and F), but was not modified in the presence of *impα1* or *impα3* mutant alleles. (G–H) Representative confocal images of ovarian follicle cells stained with an anti-Cbt antibody (green) and DAPI (blue). High-magnification images of ovarioles are shown on the right. Note that whereas the Cbt protein is detected in follicle cell nuclei (G) from wild-type flies, its nuclear localization is reduced in *impα2^D14^* mutant ovaries. Scale bars: 10 µm and 8 µm for regular and high-magnification images, respectively.

Next, we aimed to identify the molecular mechanism that regulates Cbt nuclear import. The Cbt PNKKPRL motif presents features of classical monopartite NLSs, matching the K(K/R)X(K/R) consensus sequence required for importin-α/importin-β-based nuclear transport. Importin-α binds to NLS-bearing proteins and functions as an adapter to access the importin-β-dependent import pathway [65]. In *Drosophila*, three importin-α proteins have been identified (reviewed in [66]): importin-α1 (impα1, Kapα1 or CG8548), importin-α2 (impα2, Kapα2, Pen or CG4799) and importin-α3 (impα3, Kapα3 and CG9423) [67]. *In vitro* binding studies and nuclear import assays revealed that both NLSs and protein context mediate importin-α specificity for substrate nuclear import [68]. It has been shown that most tissues express all importin-α proteins, which probably perform redundant functions. Indeed, all three importin-α proteins are required for male and female germline development [69], [70], [71], [72], [73]. To identify which importin-α is involved in Cbt nuclear transport, we first searched in the BioGrid, DrosID and DpiM interaction databases for reported interactions between importin-α proteins and Cbt [74], [75], [76]. Because no known interactions were found, other experimental approaches were used to determine possible functional relationships between Cbt and each of these proteins. First, we performed genetic interaction assays using the rough eye phenotype caused by Cbt overexpression with the *sev*-Gal4 driver [1] (Figure 7B). Using this assay, we tested whether dosage reduction of any of the *impα* genes was able to dominantly modify that phenotype. Crosses of a recombinant *sev*-Gal4>UAS-*Cbt_FL_* line with *impα1*, *impα2* and *impα3* mutant alleles (*impα1^G4113^*, *pen^EY0909^* and *impα3* ^CG397^, respectively) were performed, and the progeny were scored for eye roughness modification. Although no modification was observed with the *impα1* or *impα3* mutant alleles (Figure 7C, E), there was a mild enhancement of the eye phenotype when *impα2* function was reduced (Figure 7D). We validated this interaction using an independent *impα2* allele (*impα2^D14^*, Figure 7F). This result suggested that Cbt and Impα2 are functionally related. To confirm these results, we used UAS-*impα* RNAi lines to deplete Impα expression in larval salivary glands and then assessed whether Cbt nuclear localization was disrupted. A similar approach was previously applied to demonstrate that the Naked cuticle (Nkd) protein requires Impα3 for nuclear localization [77]. Immunostaining of salivary glands from *71B*>*impα1^RNAi^*, *71B*>*impα2^RNAi^* and *71B*>*impα3^RNAi^* larvae revealed no changes in Cbt nuclear localization (Figure S3A–D). However, only the *impα3^RNAi^* line has been shown to reduce Impα3 immunoreactivity [77].

Previous work has shown that *impα2* is expressed during embryonic development in the male and female germline [69], [70], [78], the nervous system [79] and larval muscles [44] and may be involved in cell proliferation and cell cycle progression [80], [81]. However, it is not required for general development or cell viability because *impα2* mutants survive to adulthood, possibly due to functional redundancy with other importins. Therefore, to determine whether Impα2 is required for nuclear Cbt import, we analyzed Cbt expression in the larval central nervous system (CNS) and in ovaries of wild-type and *impα2^D14^* mutants. Previously, we and others described that Cbt was a maternal factor that was expressed in the CNS during embryonic development [8], [13]. Our results show that Cbt nuclear localization is not altered in the larval brain hemispheres of *impα2* mutants (Figure S3E–F). In the ovary, Cbt is normally localized to the nuclei of follicular cells in the germarium. We observed that the nuclear localization of Cbt in these cells was significantly reduced in *impα2* mutants (Figure 7G–H). These results indicate that Impα2 may be involved in Cbt nuclear import in ovaries but not the larval CNS and suggest that no functional redundancy exists between Impα2 and the other importins during oogenesis, as previously proposed [70]. However, our results can not exclude the possibility that other Impα proteins may be required for Cbt nuclear localization in other tissues. This is supported by the fact that Cbt has a complex expression pattern during *Drosophila* embryogenesis [13].

## Discussion

Cbt is an evolutionarily conserved C_2_H_2_ zinc finger TF involved in the regulation of different developmental processes in *Drosophila* [1], [2], [3], [4], [5], [6], [7], [8], [9], [10], [12], [13]. Indeed, it is the *Drosophila* ortholog of the vertebrate TIEG proteins, which belong to the Sp1-KLF family of TFs) [14], [16]. However, little is known about the molecular mechanisms that regulate Cbt function. To fully characterize the function of a TF, it is important to identify at least three domains in its sequence: the DBD, the NLS and the transcriptional regulatory domain(s). The aims of the present study were to determine its role in transcriptional regulation as well as characterize in detail the molecular mechanisms of Cbt nuclear import by identifying the relevant functional domains. Our experiments were also designed to test the functional conservation between Cbt and its vertebrate orthologs, based on previous results obtained from functional studies of the TIEG proteins. Although it is generally accepted that evolutionarily conserved sequences will perform the same molecular function, this is not always true, and evidence for functional conservation must come from functional studies and not from sequence similarity analyses.

### Transcriptional repressor/activator activity of Cbt

In the present study we have demonstrated for the first time that Cbt acts as a transcriptional repressor in *Drosophila* S2 cells. However, previous studies suggested that it might function as a transcriptional activator. We first showed that *dpp* expression was downregulated at the leading edge of the lateral epidermal sheets in *cbt* mutant embryos during dorsal closure [1]. Furthermore, it has been recently shown that Cbt acts as positive regulator of TGF-β signaling during wing imaginal disc development [12]. This latter study suggested that the function of Cbt as a transcriptional activator was consistent with the fact that repressor domains identified in the TIEG proteins (R1, R2 and R3 domains) were not conserved in Cbt [12]. However, our results clearly show that Cbt acts as a transcriptional repressor in S2 cells and that this activity is mediated in part by a conserved SID located in its N-terminal region. This domain was found within the R1 region of the TIEG proteins and was shown to be essential for human and murine TIEGs-mediated transcriptional repression in cell culture [31], [32]. It is interesting to note that the putative SID in the Cbt protein is also located in an α-helix but in a different part of the protein. In addition, we found that the REP1 sequence located in the C-terminal region of Cbt could also account for its transcriptional repressor activity. Supporting this observation, the REP1 sequence contains charged, hydrophobic A and P residues, as has been shown for other transcriptional repressor domains [82], [83]. However, no information about similar sequences in TIEGs or other repressor proteins has been found in the literature. Interestingly, the C-terminal region of murine TIEG3 protein presents transcriptional activator activity, althought the domain(s) responsible of that function has not been characterized yet [32]. Transcriptional repression is crucial for the regulation of gene expression and morphogenesis. Hairy-related proteins play critical roles during development by repressing target genes at multiple stages of neurogenesis [84]. Similarly, early patterning of the *Drosophila* embryo requires multiple genes encoding transcriptional repressor proteins [83], [84], [85].

TIEG proteins were originally described as transcriptional repressors, but several studies have demonstrated that these proteins and other Sp1/KLF family members can be repressors or activators depending on the cellular and binding site context [31], [32], [33], [34], [35]. Indeed, it has been shown that phosphorylation of S/T residues adjacent to the SID may disrupt the mSin3A interaction, thus inhibiting TIEG2 repressor activity [86]. Besides, the KLF13 protein presents a SID overlapping with an activation domain, and its activator/repressor activity depends on the acetylation state of its DBD or its target promoters [87]. Although we do not know whether this double function is conserved in the *Drosophila* Cbt protein, it is also interesting to consider that the SR region is conserved in most of the *Drosophila* Cbt orthologs (Figure S2). SR domains have been shown to be involved in transactivation, e.g., in the v-Rel protein [88]. Maybe phosphorylations in S/T residues within the SR domain could be involved in the regulation of the transcriptional activity of Cbt. Moreover, it is important to note that despite the high similarity in the zinc finger domain between Cbt and the TIEG proteins, our results show that this region is not able to activate transcription as it does in the murine TIEG3 protein [31], [32]. Further experiments are necessary to determine whether Cbt can act as a transcriptional activator in *Drosophila*.

### Cbt regulates its own expression: negative feedback?

We also show that Cbt is able to recognize its own promoter and negatively regulate its own expression in S2 cells. This report is the first of a direct Cbt target. These data are supported by ChIP-on-chip assay results in which Cbt was found to bind to its promoter region (Figure S1). Negative feedback loops are used to regulate the levels of signaling molecules and contribute to signal homeostasis. In many cases, the molecular component that executes the feedback-mediated inhibition is transcriptionally targeted by the pathway that it regulates. This mechanism ensures an interdependence of signaling activity and feedback regulation and is often viewed as an inherent means of downregulating signaling pathways during development [89], [90]. One interesting observation that is consistent with the existence of Cbt negative feedback is that *cbt* overexpression in different tissues during embryonic dorsal closure, where it acts downstream of the JNK pathway, does not cause embryonic lethality [1] (Y.B and N.P, unpublished results), as occurs when other components of that pathway are overexpressed [91]. Cbt likely acts to negatively regulate its own expression and modulate JNK signaling levels. This negative feedback is also consistent with a role for Cbt in regulating circadian rhythms [5], as most transcriptional circadian regulators have a strong transcriptional effect (often direct) on their own synthesis in both mammals and *Drosophila* [92], [93], [94]. Interestingly, several TFs of the KLF family can regulate their own expression as well as the expression of other family members. KLF4, for example, can activate its own expression in the intestinal epithelium, while KLF5 represses KLF4 expression through competitive interaction with the same cis-element [95]. Currently, no evidence of such an autoregulatory mechanism has been demonstrated in vertebrate TIEG proteins. Several studies are being performed to identify other direct Cbt target genes and to further analyze how they are transcriptionally regulated by this protein.

### Molecular mechanism of Cbt nuclear import

NLSs of proteins belonging to the KLF family of TFs are diverse in different organisms although most are either within or nearby their DBDs [32], [38], [39], [96]. Indeed, the murine TIEG3 protein contains a bipartite NLS within the zinc finger region conserved in other TIEG proteins in mice and humans [31], [32]. In this work, we demonstrate that Cbt nuclear import is mediated by a monopartite NLS (PNKKPRL) located within the N-terminal region of the protein. This NLS is not conserved in vertebrate KLF family members but is present in insect Cbt orthologs as well as in other unrelated proteins from invertebrates and vertebrates. We also demonstrate that this NLS is functional in *S. exigua* Sec301 cells, suggesting that it is probably functional in all insect Cbt orthologs. Interestingly, Cbt also contains a second NLS, which resembles that described in the TIEG3 protein [31], [32]. Importantly, experiments investigating Cbt localization in hamster CHO-K1, *Drosophila* S2 and *S. exigua* Sec301 cells confirmed that this second NLS is functional in mammalian but not insect cells. This finding clearly demonstrates that protein sequence conservation among different species does not always indicate functional conservation and indicates that additional factors such as cellular context are also important and must be considered when ascribing molecular functions to certain sequences. A similar situation has been found in the *Aspergillus nidulans* HapB protein, a subunit of CCAAT-binding factor [97]. A likely explanation for the results obtained in our studies is that in insects, the sequence at the N terminus of the Cbt protein is recognized as an NLS by the importin-α/importin-β-based nuclear transport machinery, whereas that contained in the second and third finger domains is not [65].

Experiments using β-GalCbt fusion proteins also demonstrated the relevance of the central K residues (K73 and K74) in Cbt nuclear import as well as the existence of putatively critical residues, such as the flanking P, N, P, R and L residues and maybe residues within the C3 region (Figures 5 and S2). Nuclear transport can be regulated at multiple levels, via a diverse range of mechanisms that include (1) accessibility or masking of the NLS and availability of import factors; (2) existence of cytoplasmic or nucleoplasmic retention signals; (3) regulation of the NLS affinity for its import receptor, e.g., by phosphorylation; (4) regulation of nuclear pore complex permeability; and (5) possible regulation of cargo affinity to the hydrophobic central channel. Among these mechanisms, post-translational modification of proteins through phosphorylation/dephosphorylation is the best understood mechanism regulating nuclear transport (reviewed in [42]). To analyze this possibility, we used the NetPhos software [98] to perform *in silico* predictions of putative S, T and tyrosine (Y) phosphorylation sites around the Cbt NLS and to identify the kinases putatively responsible for the predicted phosphorylation events. These analyses showed that although the _71_PNKKPRL_77_ sequence is not probably modified, the conserved S59 and T61 residues could be phosphorylated by Casein Kinase II (CKII) and the T85 amino acid could be targeted by the p38 Mitogen-activated protein kinase (p38 MAPK) (Figures S2 and S4). Because the β-GalCbt_1–76_ fusion protein, which lacks S83 and T85, can be translocated to the nucleus (Figure 5D), the S59 and T61 residues are better candidates for phosphorylation sites that affect Cbt nuclear transport. Interestingly, we found that the S59 and T61 residues near the PNKKPRL sequence were conserved in the twelve *Drosophila* species analyzed (Figure S2). This finding could explain why the β-Gal protein is not efficiently transported to the nucleus when fused to the PNKKPRL sequence (Figure 5H–I). Similar results have been reported for proteins fused to the SV40 NLS and in the KLF8 protein [99], [100]. Phosphorylation of residues flanking the NLS can affect nuclear trafficking in different ways, e.g., enhancing the binding affinity of importins to cargo or enhancing the docking of cargo to the nuclear pore complex as well as causing conformational changes that expose the NLS to the protein surface (reviewed in [61]). Additional experiments will be necessary to confirm that Cbt nuclear translocation is regulated by the phosphorylation/desphosphorylation of these residues.

Regarding Cbt, previous studies revealed that this protein is expressed as a Pumilio target in central and peripheral nervous system axons [13] and probably synapses [101] of the *Drosophila* embryo. Although we have not yet identified a nuclear export signal (NES) in the Cbt sequence (data not shown), these findings suggest that Cbt nuclear import might be tightly regulated either by post-translational modifications (such as phosphorylation of S59 and T61 residues) or conformational changes that prevent NLS recognition by importins. Cbt has also been reported to be involved in circadian rhythms [5], a process in which the control of nuclear trafficking has been demonstrated [102]. Indeed, several clock proteins contain NLSs that facilitate their cellular trafficking [20], [103]. This control of trafficking is key for the generation and maintenance of robust and coherent circadian rhythms. Although the mechanisms of nuclear transport regulation mentioned above have been previously described for other proteins (reviewed in [104]), further analyses will be required to confirm whether they also regulate Cbt nuclear trafficking

Finally, genetic interaction assays and Cbt immunostaining in *impα* mutants suggest that the Impα2 protein may interact with Cbt, probably recognizing the PNKKPRL sequence, and seems to be required to transport Cbt to the nucleus in several tissues, including the *Drosophila* ovary, where a reduction of Cbt nuclear localization was observed in *impα2* mutant flies. In support of this hypothesis, the human BRCA protein contains an NLS similar to the one detected in Cbt; this NLS is recognized by the Impα2 protein and is responsible for the protein's nuclear localization [64]. These data suggest that the nuclear transport mechanism mediated by the PX(K/R)KX(R/L) sequence may be conserved between vertebrates and invertebrates. *Drosophila* Impα2 was recently shown to be involved in Frizzled regulation in muscle and in the central and peripheral nervous systems of embryos and larvae [44], [79]. However, we do not exclude the possibility that other Impα proteins (α1 or α3) may be involved in Cbt nuclear transport in other tissues because Cbt presents a ubiquitous expression pattern at embryonic and larval stages [13]. Co-immunoprecipitation assays will be necessary to determine whether other importin proteins interact with Cbt in different contexts.

## Supporting Information

Figure S1 **Cabut binds to its own promoter region.** (A) Integrate Genome Browser (IGB) [105] overview of the *cabut* genomic region on chromosome 2L identified in the ChIP-on-chip analysis. From top to bottom: signal represents the log_2_ of normalized ratios of IP/input, p-value is on a −10log_10_ scale (based on Wilcoxon test), coordinates of the genomic fragment and structure of the *cabut* gene. (B) Nucleotide sequence of the *cabut* Prom1-2 region. The GC-rich sequences that might be recognized by the Cbt protein are boxed.
(TIF)

Figure S2 **Multiple alignment of Cabut orthologs from twelve** ***Drosophila*** **species, showing conserved domains and secondary structure.** C1, C2, C3, C4/Rep1 and C5 boxes contain conserved regions in the N and C termini of *Drosophila* Cbt proteins; the NLS box indicates the position of the sequence required for Cbt nuclear localization in *D. melanogaster*. The AAEVAL and Rep1 boxes mark the transcriptional repressor motifs identified in this work. The positions of the serine-rich (SR) region and the three zinc fingers (Zn1, Zn2 and Zn3) are also indicated. Black diamonds mark the position of S and T residues that are potentially phosphorylatable (according to NetPhos program) and putatively required for regulation of Cbt nuclear import. Red arrows and yellow helixes indicate the positions of predicted β-sheet motifs and α-helixes, respectively. The secondary structure topology was obtained using the SWISS-MODEL program.
(TIF)

Figure S3 **Immunohistochemical detection of the Cabut protein in** ***importin-α*** **mutants.** (A–C) Immunostaining of salivary glands from (A) wild-type, (B) *71B*>*impα3^RNAi^*, (C) *71B*>*impα1^RNAi^*, and (D) *71B*>*impα2^RNAi^* third instar larvae with anti-Cbt (red) and anti-Lamin (green) antibodies. Note that Cbt was still detected in the nuclei of salivary gland cells (arrow) and fat cells (arrowhead) in all genotypes. Scale bar: 15 µm. (E–F) Immunostaining of brain hemispheres from (E) wild-type and (F) *impα2^D14^* mutant larvae with an anti-Cbt (green) antibody. Note that Cbt nuclear localization was not reduced in brains of *impoα2* mutants. Scale bar: 10 µm.
(TIF)

Figure S4 ***in silico*** **prediction of putative phosphorylatable residues in the Cabut sequence.** The positions of S and T residues predicted to be susceptible to phosphorylation by different kinases (indicated in pink: CK II and p38-MAPK) and adjacent to the PNKKPRL sequence (whose position is marked by a red arrow) are shown. Putative phosphorylatable residues and responsible kinases were determined using the NetPhos and NetPhosK programs, respectively.
(TIF)
