# Supplementary figures and images for: Transcriptional Activity and Nuclear Localization of Cabut, the Drosophila Ortholog of Vertebrate TGF-β-Inducible Early-Response Gene (TIEG) Proteins
Source: PLoS One. 2012 Feb 16;7(2):e32004. doi: 10.1371/journal.pone.0032004 (PMC3281117; doi:10.1371/journal.pone.0032004)

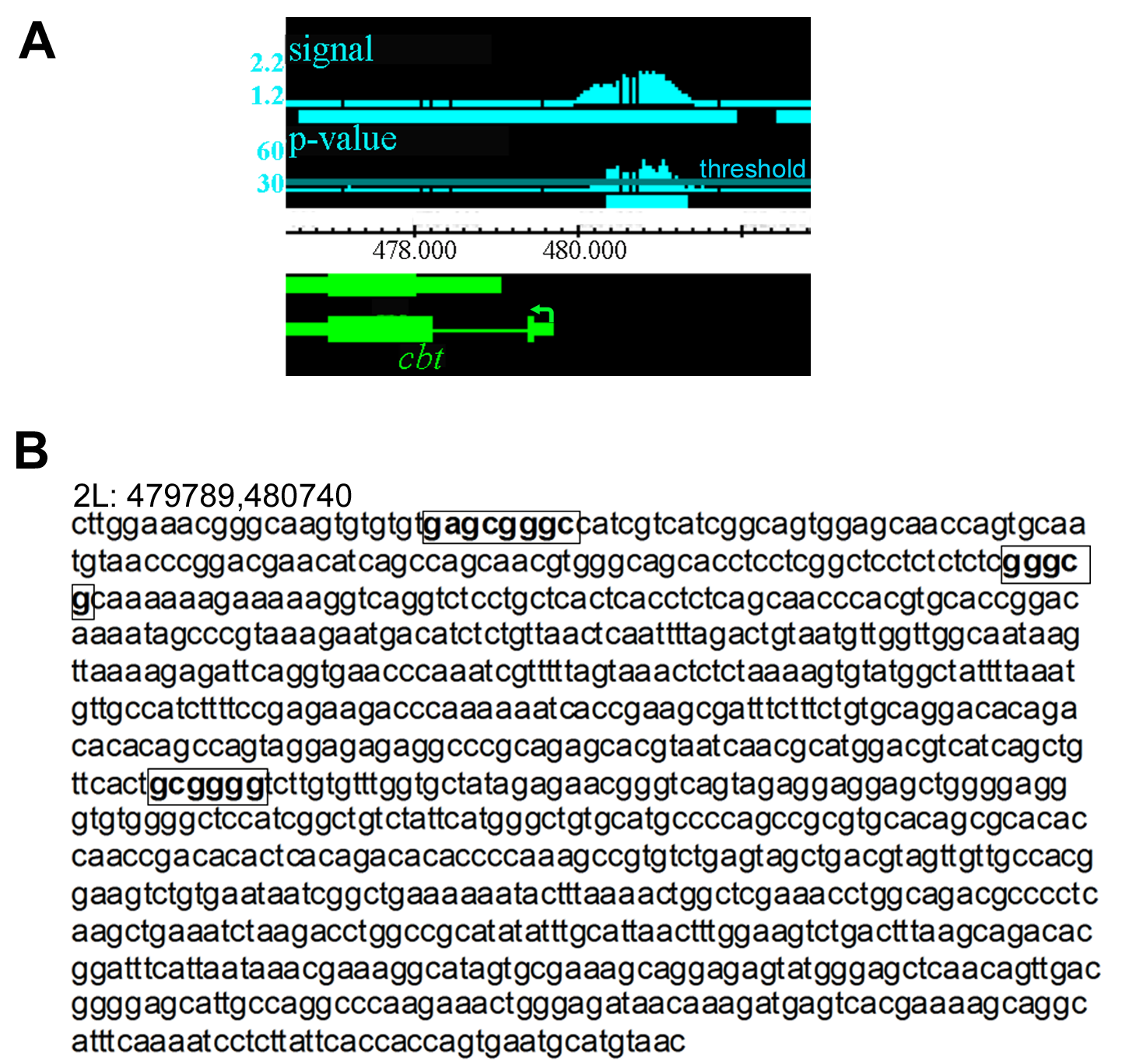

Supplement: Figure S1 — Cabut binds to its own promoter region. (A) Integrate Genome Browser (IGB) [105] overview of the cabut genomic region on chromosome 2L identified in the ChIP-on-chip analysis. From top to bottom: signal represents the log2 of normalized ratios of IP/input, p-value is on a −10log10 scale (based on Wilcoxon test), coordinates of the genomic fragment and structure of the cabut gene. (B) Nucleotide sequence of the cabut Prom1-2 region. The GC-rich sequences that might be recognized by the Cbt protein are boxed. (TIF) [file pone.0032004.s001.tif]

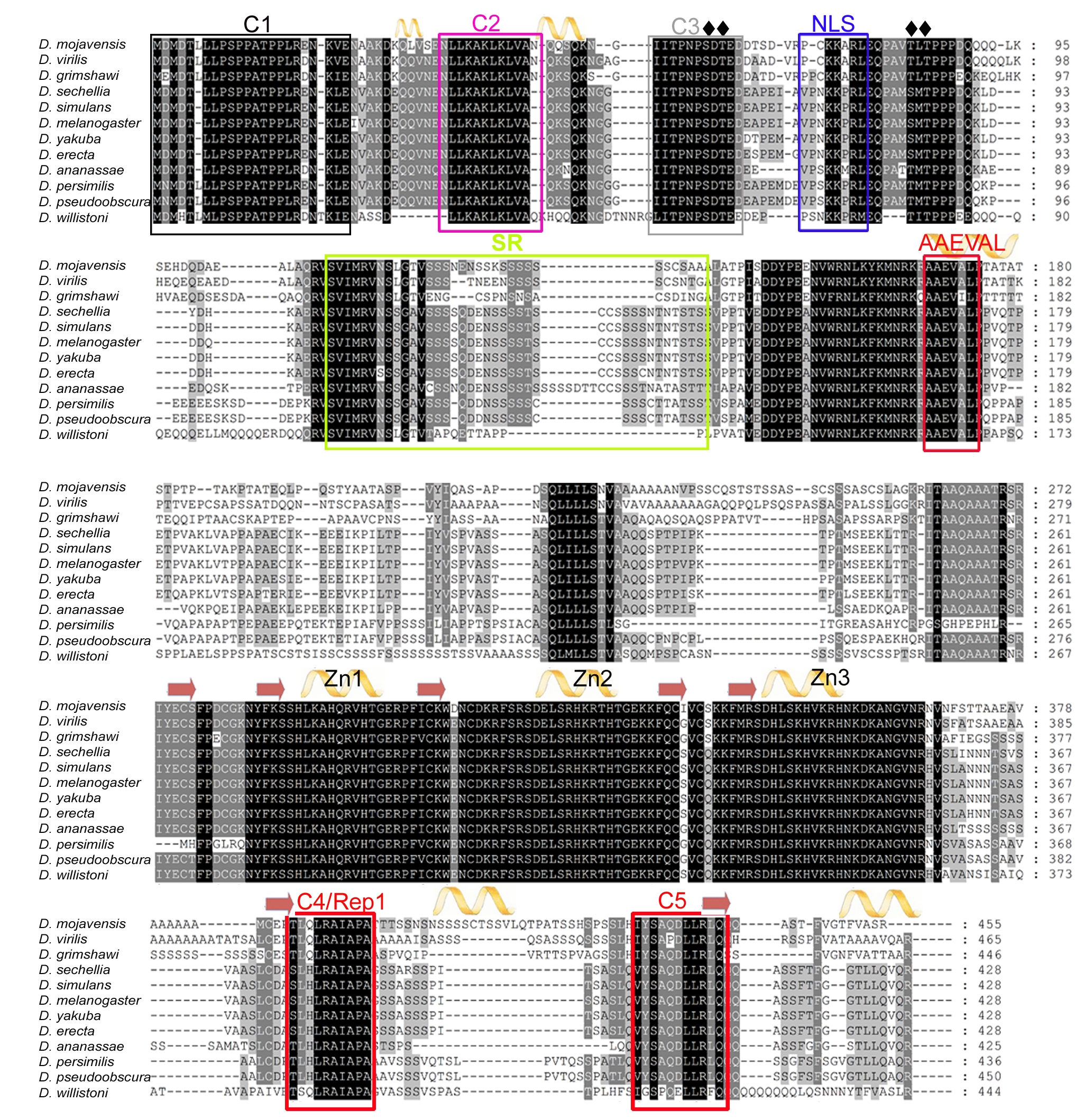

Supplement: Figure S2 — Multiple alignment of Cabut orthologs from twelve Drosophila species, showing conserved domains and secondary structure. C1, C2, C3, C4/Rep1 and C5 boxes contain conserved regions in the N and C termini of Drosophila Cbt proteins; the NLS box indicates the position of the sequence required for Cbt nuclear localization in D. melanogaster. The AAEVAL and Rep1 boxes mark the transcriptional repressor motifs identified in this work. The positions of the serine-rich (SR) region and the three zinc fingers (Zn1, Zn2 and Zn3) are also indicated. Black diamonds mark the position of S and T residues that are potentially phosphorylatable (according to NetPhos program) and putatively required for regulation of Cbt nuclear import. Red arrows and yellow helixes indicate the positions of predicted β-sheet motifs and α-helixes, respectively. The secondary structure topology was obtained using the SWISS-MODEL program. (TIF) [file pone.0032004.s002.tif]

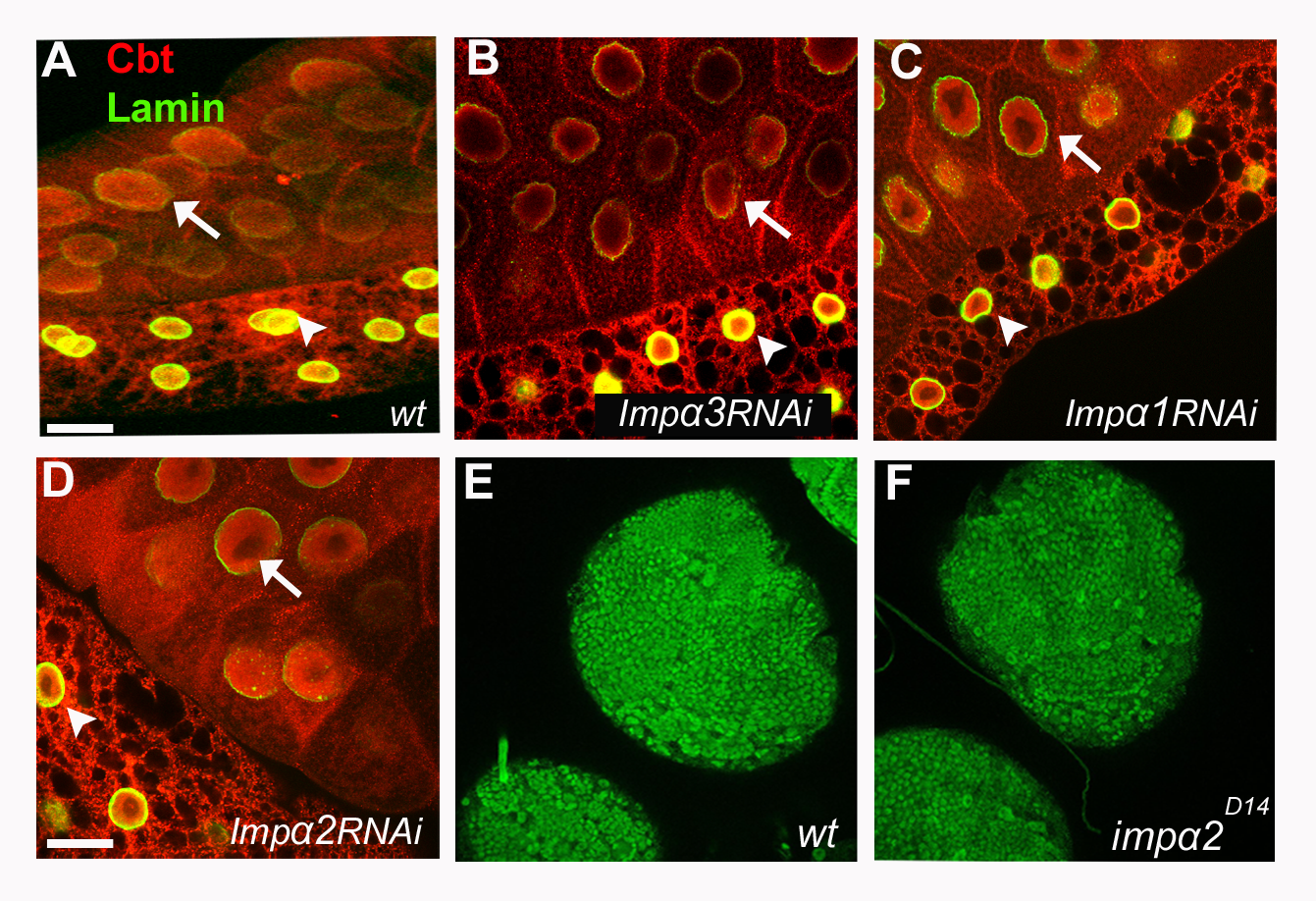

Supplement: Figure S3 — Immunohistochemical detection of the Cabut protein in importin-α mutants. (A–C) Immunostaining of salivary glands from (A) wild-type, (B) 71B>impα3RNAi, (C) 71B>impα1RNAi, and (D) 71B>impα2RNAi third instar larvae with anti-Cbt (red) and anti-Lamin (green) antibodies. Note that Cbt was still detected in the nuclei of salivary gland cells (arrow) and fat cells (arrowhead) in all genotypes. Scale bar: 15 µm. (E–F) Immunostaining of brain hemispheres from (E) wild-type and (F) impα2D14 mutant larvae with an anti-Cbt (green) antibody. Note that Cbt nuclear localization was not reduced in brains of impoα2 mutants. Scale bar: 10 µm. (TIF) [file pone.0032004.s003.tif]

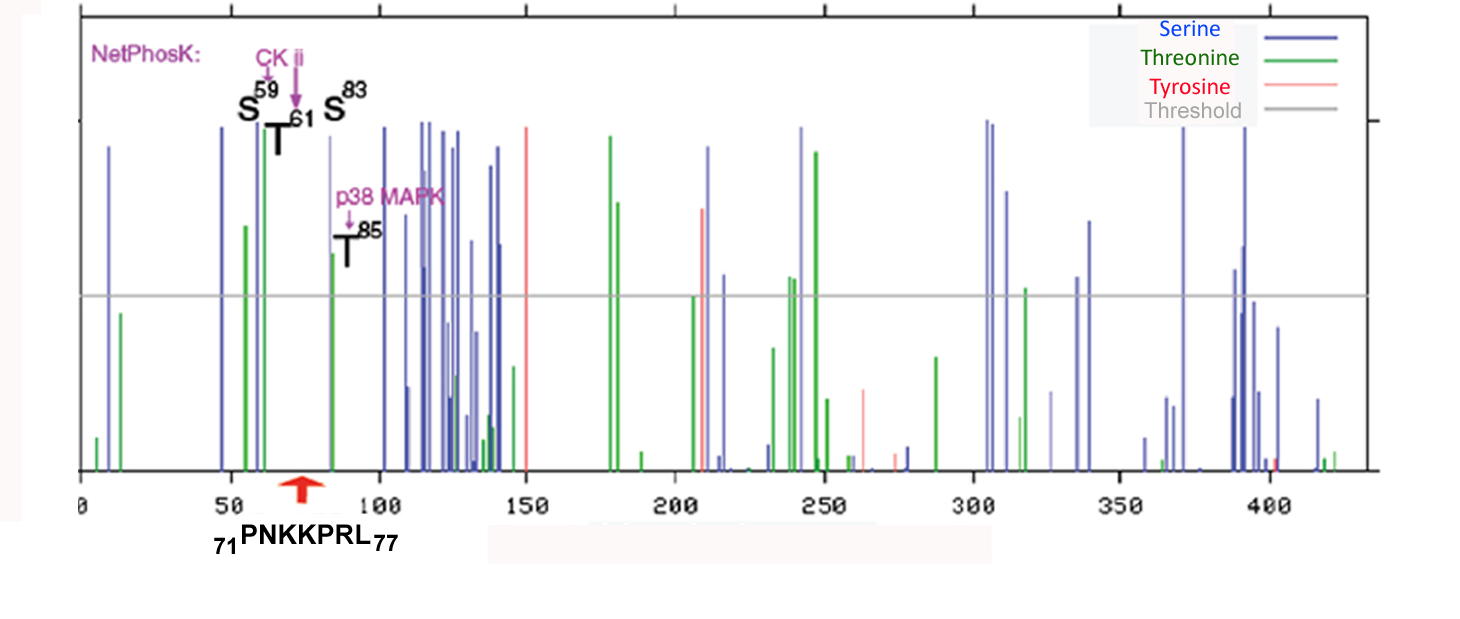

Supplement: Figure S4 — in silico prediction of putative phosphorylatable residues in the Cabut sequence. The positions of S and T residues predicted to be susceptible to phosphorylation by different kinases (indicated in pink: CK II and p38-MAPK) and adjacent to the PNKKPRL sequence (whose position is marked by a red arrow) are shown. Putative phosphorylatable residues and responsible kinases were determined using the NetPhos and NetPhosK programs, respectively. (TIF) [file pone.0032004.s004.tif]
